# Chronic stress boosts systemic inflammation and compromises antiviral innate immunity in *Carassius gibel*

**DOI:** 10.3389/fimmu.2023.1105156

**Published:** 2023-02-06

**Authors:** Caijiao Dai, Jianduo Zheng, Lin Qi, Ping Deng, Mengke Wu, Lijuan Li, Junfa Yuan

**Affiliations:** ^1^ Department of Aquatic Animal Medicine, College of Fisheries, Huazhong Agricultural University, Wuhan, China; ^2^ National Aquatic Animal Diseases Para-reference Laboratory, Huazhong Agricultural University (HZUA), Wuhan, China; ^3^ Hubei Engineering Research Center for Aquatic Animal Diseases Control and Prevention, Huazhong Agricultural University, Wuhan, China; ^4^ Department of Consultation, Tianbin Ruicheng Environmental Technology Engineering Co., LTD, Tianjin, China; ^5^ Fisheries Science Research Institute, Wuhan Academy of Agricultural Sciences, Wuhan, China

**Keywords:** inflammation, chronic stress, stress, CyHV-2, antiviral immune

## Abstract

It is generally considered that stress causes decreased immune function and render fish vulnerable to infection and diseases. However, the molecular mechanisms between stress responses and susceptibility to infections, especially viral diseases, in fish remain unknown. Understanding and monitoring the biological consequences and mechanisms underlying stress responses in fish may contribute to the improvement of animal welfare and production efficiency. In this study, long-term exposure to a variety of stressors, including chasing, overcrowding, restraint stress, and air exposure mimicking chronic stresses, in aquaculture practices was conducted in *Carassius gibel* to investigate the consequences of chronic stress on inflammation and antiviral capability. With the continuation of stimulation, experimental fish gradually became insensitive to the stress of net chasing and feeding with the accompaniment of upregulated gene expressed in the HPI axis and elevated levels of stress hormones. As expected, stress-induced hyperglycaemia with a decrease in the insulin signaling pathway and altered gene expression in glycolysis and gluconeogenesis, suggesting the disturbance of glycometabolism. Importantly, a link between intestinal homoeostasis and systemic low-grade inflammation in stressed *C. gibel* was observed, implying crosstalk among the brain, intestine, and other organs. Furthermore, the compromised antiviral capability with impaired antiviral innate immunity in stressed fish was confirmed by RNA sequencing and infection with *Cyprinid herpesvirus 2* (CyHV-2), promoting the understanding of enhanced susceptibility to viral infection in stressed fish.

## Introduction

Similar to other vertebrate, stress triggers vastly diverse repercussions on physiological function including metabolic changes and immune alterations in fish (1–3). It is generally considered that stress causes decreased immune function and render fish vulnerable to infection and diseases (4) However, the intrinsic interaction between stress responses and the occurrence of infections remains obscure. The main stressors in aquaculture derive from elevated rearing densities, decreased dissolved oxygen, elevated levels of ammoniacal nitrogen and nitrite, thermal fluctuations, diet, and invasion of pathogens (5, 6). Stress can also be a consequence of transportation, sorting, handling and confinement (7–9). Anti-stress management has become an important routine work under current aquaculture practices and contributes to the decrease in opportunistic infections (10–12). Understanding and monitoring the biological mechanisms underlying stress responses in fish may alleviate the adverse effects of stress through selective breeding and changes in management practices to improve animal welfare and production efficiency.

Acute stress refers to a short-term and adaptive state, accompanied by endocrine and neurotransmitter release (13, 14). In aquaculture practices, fishing exercise during traditional fry breeding in Southeast Asian countries exemplifies the usage of moderate acute stress to adapt farming handling, including transportation and sorting. In contrast, chronic stress involves repeated or prolonged action of stressors (15). Notably, farmed fish are accompanied by diverse stresses during their lifetime; therefore, chronic stress is one of the dominant threats to farmed fish (12). Chronic stress, including long-term overcrowding and repeating handling, were investigated in grass carp, gilthead seabream, Senegalese sole, brown trout and other species (16, 17). Most of these studies concentrated the correlation between cortisol levels and increased susceptibility to diseases. However, cortisol has a biphasic effect on the immune system (4). Furthermore, effects of chronic stress on cortisol level are a controversial issue (18–20). For instance, crowding stress in rainbow trout resulted in elevated plasma cortisol levels up to 6 days, but values returned to basal levels after 10 days. While rainbow trout subjected to daily handling stress for 10 weeks did not show chronic elevation of total plasma cortisol levels. The complexed feedback effect of cortisol on the activity might link with the inconsistency.

Stress is associated with alterations in the immune and inflammatory systems (21–23) Clinical and experimental investigations have demonstrated that proinflammatory cytokines (IL-1, IL-6, IL-8 and others) and acute phase proteins are increased in humans, mice, and fish with posttraumatic stress compared to healthy individuals, which affects disease progression, including stress hyperglycemia, allergic diseases, autoimmune diseases, and cancers (24–27). It is generally accepted that acute stress increases resistance to infection, while chronic stress deteriorates and increases complications of infection (20, 28–30). Studies have linked chronic stress with herpes simplex virus reactivation, tuberculosis, ulcers, and other infectious diseases in humans (5). Recent work demonstrated that depression attenuates antiviral innate immunity *via* the AVP-AH1-Tyk2 axis by regulating basal IFN signaling in patients or mice (31). Farmed fish run a risk of multiple viral diseases (32–34). Therefore, viral infection under the condition of chronic stress is considered one of the leading threats in the aquaculture industry (32). Although intensive physical and psychological stress is known to modulate immune function, mechanistic pathways linking chronic stress and antiviral immunity remain poorly understood, especially in the low vertebrates.

In this study, the effect of long-term stimulation mimicking chronic stresses in aquaculture practices on inflammation and antiviral capability was investigated in gibel carp. The link between imbalance of intestinal homoeostasis and low-grade inflammation in stressed fish was observed, suggesting crosstalk among the brain, intestine, and other organs.

## Materials and methods

### Virus and fish

The CyHV-2 isolate of SY-C1 was used in this study (35). The experimental fish with average weight of 100 ± 4.31 g and average length of 10.32 ± 1.0 cm were provided by a local commercial farm (Wuhan, Hubei, China). Before the experiment, they were temporarily housed for 14 days in a holding aquarium (0.8 m^2^× 1.2 m) at 23-25°C, containing aerated, carbon-filter, dechlorinated tap water, and a natural photoperiod. In order to ensure the experimental effect, 15 fish per group were maintained in a holding aquarium. All fish used in this study were maintained and cared for HZAU in accordance with Institutional Animal Care and Use Committee guidelines that were approved by the Scientific Ethic Committee of Huazhong Agricultural University (HZAUFI-2018-016).

### Chronic stress treatment

Gibel carp were subjected to a variety of chronic stressors including chasing, overcrowding, restraint stress, and air exposure. Experimental fish were exposed to a series of stressors twice per day for 21 days. The stressors were conducted as follows: chasing by a net for 1 min, overcrowding with 15 fish in a net for 2 min, and then 1-min air exposure (by lifting the fish from their tanks by the net and leaving in the air). The duration and sequence of the indicated stressors were constant, but the beginning time of stress was changed daily to avoid habituation to the stressors. Aeration and temperature were maintained throughout the stressor procedure. Control fish were maintained in the same room without visual contact to the stressed groups throughout the experiment. During the period of stressing, the fish were fed twice daily with formulated feed. Six arbitrary individuals from control and treated fish were sampled every 7 days, and samples of each phase were collected at 2 h after they were stressed. For sampling, blood and tissues, including the liver, trunk kidney, spleen, and midgut, as well as the hypothalamus and pituitary gland, were collected, snap-frozen in liquid nitrogen and kept at -80°C until further analysis.

### Infection assay with CyHV-2

Healthy fish were infected intraperitoneally with 0.1 mL virus stock containing 1×10^5.5^ copies/mL CyHV-2 (0.1 × LD50), as descripted previously (35) and named as Group A. Briefly, 1 g of kidney tissue from CyHV-2-infected *Carassius gibel* were pooled and homogenized in 10 mL of TN buffer (50 mmol/L Tris–HCl, 100 mmol/L NaCl, pH 7.4), followed by centrifugation at 5000 × g for 10 min at 4°C. Subsequently, the supernatant was filtered through a 0.45-µm acetate cellulose filter and subjected for virus quantitate by qPCR. For control, a total of 30 stress-treated fish were injected intraperitoneally with 0.1 mL of TN buffer (Group B). Another group of 30 stressed-fish were infected the same does of CyHV-2 with group C. During the period of infection, all fish were kept at 23-25°C and mortality was recorded daily. The spleen and trunk kidney tissues were sampled from moribund fish for CyHV-2 quantification.

### Behavioral experiment

Six gibel carp (100 ± 4.31g) were selected from stressed group and control group respectively and placed in a white opaque container (100 cm × 70 cm) for behavioral experiment. For dark adaptation, the containers were covered with a black net for 90 min before behavioral testing. The swimming behavior of the selected fish was recorded by the camera above the white opaque container. The swimming time was calculated from the moment of pulling the black net and ended until all fish calmed down according to the video. As for the struggling time, 6 fish were caught in a net and exposed to air. The struggling time was calculated from the moment of air exposation and ended until the fish stopped struggling. All the recorded time was showed as the means ± SEMs for three independent tests.

### RNA extraction, synthesis of cDNA, and quantitative PCR assay

Total RNA from 0.1 g of the indicated tissues was extracted using TRIzol (Cwbio, China) according to the manufacturer’s instructions. The concentrations and quality of RNA were analysed by a NanoDrop 2000 (Thermo Scientific, USA). According to the manufacturer’s suggestion, HiScript^®^ reverse transcriptase was used to process total RNA into cDNA (Vazyme Biotech, China), and quantitative PCR was conducted in 20 µl reactions containing 10 µl of AceQ^®^ qPCR SYBR Green Master Mix and 0.5 µl of each indicated primer. Amplification reactions were carried out under the following cycle conditions: 96°C for 3 min; 35 cycles of 96°C for 30 s, 56°C for 60 s, and 72°C for 90 s; and 72°C for 10 min. The oligonucleotides used in this study are detailed in **Table S1**. Specifically, β-actin was used as an endogenous control to normalize gene expression and the data were calculated using the 2^−ΔΔCt^ method (36).

### Quantitation of serum cortisol, insulin, blood glucose and C-reactive protein

Serum cortisol contents were determined by a commercial ELISA kit (Solarbio, Shanghai, China) according to the manufacturer’s instructions. An insulin level assay was conducted using a commercial ELISA kit (Solarbio, Shanghai, China). Fasting blood glucose was tested by an ELISA kit (Solarbio, Shanghai, China) according to the manufacturer’s instructions. C-reactive protein was measured by fish high sensitivity C-reactive protein ELISA kit (Huzheng. Shanghai, China) according to the manufacturer’s instructions.

### Intestinal microbiota analysis

Midgut tissues containing contents were sampled from stress and control fish and their DNA was extracted using a Fast DNA SPIN kit (QIAGEN, USA). The V3-V4 region of the 16S rRNA gene was amplified using the primer pair of 338F (5′-ACTCCTACGGGAGGCAGCA-3′) and 806R (5′GGACTACHVGGGTWTCTAAT-3′) and the products were subjected to high-throughput sequencing on the HiSeq2500 platform (Personalbio, Shanghai). After raw reads with low quality were removed by QIIME2 and Vsearch, species annotation was performed based on the Greengenes database (Release 13.8, http://greengenes.secondgenome.com/). Alpha diversity analysis, including the Chao1 index, Shannon index, and Simpson index, was carried out by QIIME2 (2019.4). A beta diversity analysis was performed by distance matrices run through the Bray-Curtis distance and unweighted UniFrac distance based on a PCoA analysis. The 16S rRNA sequencing data have been deposited in GenBank with accession PRJNA 891274.

### RNA sequencing analysis

After total RNA was qualified and quantified, mRNA was purified using oligo(dT)-attached magnetic beads and then fragmented into small pieces with fragment buffer. Then, first-strand cDNA was generated using random hexamer-primed reverse transcription, followed by second-strand cDNA synthesis. After amplification and library preparation, paired-end reads were generated from an Illumina HiSeq platform. Clean reads were obtained after Raw reads containing poly-N and low-quality reads (Q > 20) were filtered. Then, the clean reads were mapped to the reference genome of gibel carp (ASM336829v1) using hisat2. Gene expression levels were calculated using RSEM (v1.2.12). Gene Ontology (GO) and Kyoto Encyclopedia of Genes and Genomes (KEGG) pathway analyses of differentially expressed genes (DEGs) were performed using DESeq2 R. The RNA-seq raw data have been deposited in GenBank with accession SAMN31799252/3.

### Statistical analysis

SPSS (Version 21.0) was used to conduct the statistical analyses and comparisons between the differences in groups. A two-tailed Student’s t test was used to determine the significance between two groups. *P* values of less than 0.05 were considered statistically significant (* *p* < 0.05).

## Results

### Sustained stimulus causes chronic stress in gibel carp

**Figure 1:**
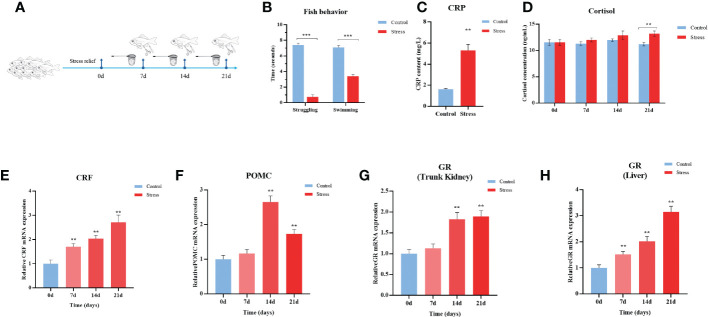
Chronic stress activates the HPI axis in gibel carp. **(A)** Schematic representation of the chronic stress model and sampling schedule. **(B)** Quantification of fish struggling and swimming behaviour after 21 days of twice daily stimulation. **(C)** ELISA analysis of serum C-reactive protein (CRP) level in gibel carp from healthy controls and stressed individuals (n = 6) after 21 days of twice daily stimulation. **(D)** ELISA analysis of serum cortisol levels in gibel carp from healthy controls (n = 6) and stressed individuals (n = 6). **(E)** RT-qPCR analysis of CRF mRNA levels in gibel carp hypothalamus from healthy controls (n = 6) and stressed individuals (n = 6). **(F)** RT-qPCR analysis of POMC mRNA levels in gibel carp hypothalamus from healthy controls (n = 6) and stressed individuals (n = 6). **(G, H)** RT-qPCR analysis of GR mRNA levels in gibel carp hypothalamus and liver, respectively, from healthy controls (n = 6) and stressed individuals (n = 6). All graphs show the means ± SEMs for individual fish, and significant differences from the control are indicated by asterisks (**P < 0.01, ***P ≤ 0.001). All graphs show the means ± SEMs for individual fish. Blue represents healthy control, and red represents stressed fish.

To mimic chronic stress in aquaculture practice, gibel carp were stimulated by net chasing, overcrowding, and air exposure twice per day for 21 days (**Figure 1A**). With the duration of stimulation, the experimental fish gradually became insensitive to the stressors of net chasing and feeding, which was manifested by the shortened struggling time response to air exposure and swimming time after light exposure (**Figure 1B**). C-reactive protein (CRP), a nonspecific marker of inflammation, significantly increased 3.26 -fold in the stress group compared with the control group (**Figure 1C**). In teleost fish, the stress response is meditated by the hypothalamic-pituitary-interrenal (HPI) axis (37). As shown in **Figures 1D–H**, the transcript levels of corticotropin-releasing factor (CRF) and proopiomelanocortin (POMC) were significantly upregulated by 2.7-fold and 1.7-fold in the hypothalamus at the end of stressing period, respectively (**Figures 1E, F**). In line with the activation of the HPI axis in stressed fish, the level of cortisol was increased after 21 d of stimulation (**Figure 1D**). Elevated transcriptional levels of GR of 3.2-fold and 1.9-fold in the liver and kidney tissues, respectively, were observed (**Figures 1G, H**). In total, fish behaviour, up-reregulated gene expression in the HPI axis, and elevated levels of stress hormone in serum indicated the stressful status of experimental gibel carp.

### Chronic stress disturbs glycometabolism in gibel carp

**Figure 2:**
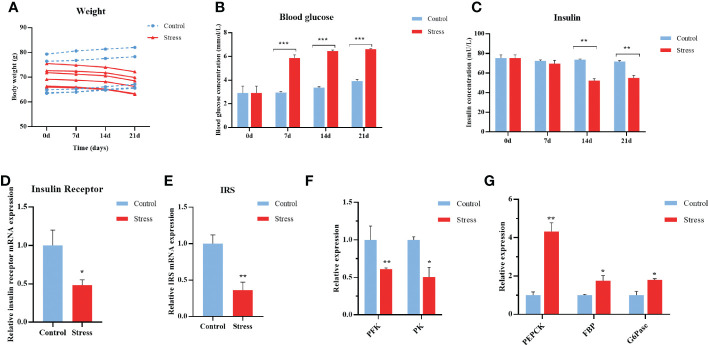
Chronic stress disturbs the glycometabolism of gibel carp. **(A)** Body weight of healthy controls and stressed fish. **(B)** ELISA analysis of fasting blood glucose levels in crucian from healthy controls (n = 6) and stressed individuals (n = 6). **(C)** ELISA analysis of serum insulin levels in gibel carp from healthy controls (n = 6) and stressed individuals (n = 6). **(D)** RT-qPCR analysis of insulin receptor mRNA levels in gibel carp livers from healthy controls (n = 6) and stressed individuals (n = 6). **(E)** RT-qPCR analysis of IRS mRNA levels in gibel carp livers from healthy controls (n = 6) and stressed individuals (n = 6). **(F)** RT-qPCR analysis of PFK and PK mRNA levels in gibel carp livers from healthy controls (n = 6) and stressed individuals (n = 6). **(G)** RT-qPCR analysis of PEPCK, FBP, and G6Pase mRNA levels in gibel carp livers from healthy controls (n = 6) and stressed individuals (n = 6). All graphs show the means ± SEMs for individual fish, and significant differences from the control are indicated by asterisks (*P < 0.05, **P < 0.01, ***P ≤ 0.001). Blue represents heathy control and red represents stressed fish.

Cortisol inhibits glucose uptake, induces lipolysis, and increases gluconeogenesis in humans and other mammals (37). Gibel carp suffering from multiple stressors showed decreased appetite, resulting in sustained weight loss when compared with the control fish (**Figure 2A**). As shown in **Figure 2B**, significantly upregulated levels of blood glucose were observed in gibel carp after they were stimulated for 7-21 d. The concentration of blood glucose in stressed individuals was changed from 2.91 mmol/L to a peak value of 6.61 mmol/L at Day 7 and remained constant from 7-21 d. Nonetheless, the contents of serum insulin in the stressed gibel carp began to decrease from the initial concentration of 75.24 mU/L at Days 0-7 to 54.95 mU/L at Days 14-21 after stimulation, suggesting the disturbance of glycometabolism. In comparison, the concentration of insulin in control carps was invariable (**Figure 2C**).

The liver is of utmost importance to glucose homeostasis by charging approximately 80% of glucose production and breakdown (38). The expression levels of insulin receptor (InsR) and insulin receptor substrates (IRS) in the liver sampled from stressed fish were downregulated by more than 50% compared with control fish (**Figures 2D, E**). Furthermore, decreased expression of glycolysis, including phosphofructokinase (PFK) and pyruvate kinase (PK) in the hepatopancreas was observed at the end of stress (**Figure 2F**). The transcript levels of gluconeogenesis rate-limiting enzymes, including phosphoenolpyruvate carboxykinase (PEPCK), fructose-1, 6-bisphosphatase (FBP), and lucose-6-phosphatase (G6Pase) were significantly upregulated (**Figure 2G**). Collectively, chronic stress impairs glycemic control in gibel carp.

### Chronic stress hampers intestinal homoeostasis in gibel carp

**Figure 3:**
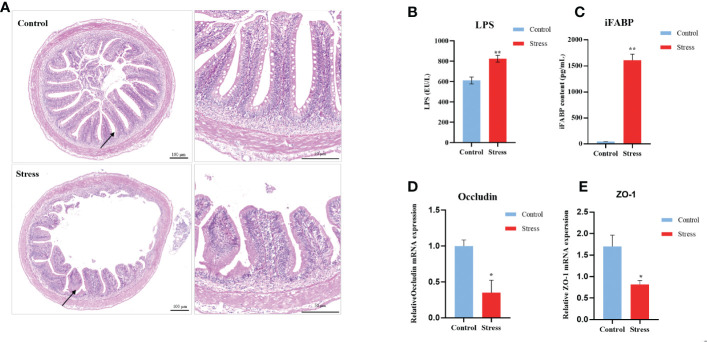
Chronic stress causes intestinal damage in gibel carp. **(A)** Clinical pathologica of intestine in healthy controls and stressed individuals (n = 2). On the left is an overall view of intestinal pathology, and the image on the right is the location specified by the black arrow on the left. **(B)** Serum levels of lipopolysaccharide (LPS) determined by ELISA from healthy controls (n = 6) and stressed individuals (n = 6).**(C)** Serum levels of intestinal fatty acid-binding protein (iFABP) determined by ELISA from healthy controls (n = 6) and stressed individuals (n = 6). **(D)** RT-qPCR analysis of occludin mRNA levels in gibel carp gut from healthy controls (n = 6) and stressed individuals (n = 6). **(E)** RT-qPCR analysis of ZO-1 mRNA levels in gibel carp guts from healthy controls (n = 6) and stressed individuals (n = 6). All graphs show the means ± SEMs for individual fish, and significant differences from the control are indicated by asterisks (*P < 0.05, **P < 0.01). Blue represents healthy control and red represents stressed fish.

Stress in humans has been confirmed to decrease mucosal barrier function in the intestine. At the final stress period, the midgut was removed for assessment of morphological changes, mucosal barrier functions, and the microbiome. Compared with healthy controls, stressed fish manifested the obvious morphological changes, including damaged even obliterated villi, decreased numbers of goblet cells, and loosened submucous layer **Figure 3A**. The serum levels of LPS and iFABP were further determined by ELISA to evaluate intestinal barrier function. The contents of LPS and iFABP were significantly increased in the stressed group compared with the control fish (**Figures 3B, C**). Additionally, chronic stress resulted in the significant downregulation of the transcript levels of the carp intestinal tight junction molecules Occludin and ZO-1. These results indicated impaired barrier function in stressed fish (**Figures 3D, E**).

**Figure 4:**
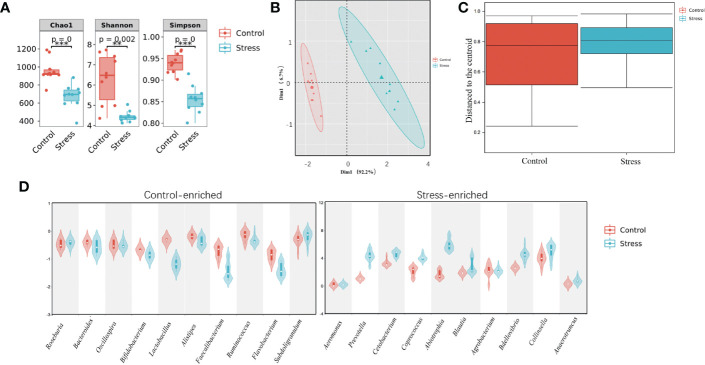
Chronic stress causes an imbalance in the intestinal microbiota in gibel carp. **(A)** The α-diversity comparison of the intestinal microbiota between healthy controls and stressed individuals (n = 10). The Chao1 index, Shannon index and Simpson index were calculated. Significant differences are indicated by asterisks (**P≤ 0.01, ***P≤0.001). **(B)** Principal component analysis (PCA) based on Bray-curtis distance were performed. **(C)** Multivariate dispersions (PERMDISP) was conducted to assess the distances from the group centroid. **(D)** Enriched microbes from healthy controls (Control, left panel, n = 10) and stressed fish (Stress, right panel, n = 10).

Intestinal microbiota has been inextricably linked with IBD and depression which is linked with chronic stress. To demonstrate the influence of chronic stress on the composition of intestinal microbiota, the midgut was sampled from control and stressed fish for 16S rRNA sequencing. In total, 9587 operational taxonomic units (OTUs) across the 10 stressed fish (stress group) and 10 control fish samples (control group) were obtained. These OTUs were classified into 23 phyla and 103 species (**Table S2**). Chronic stress changed the relative abundance of intestinal flora. As showed in **Figure 4A**, the α-diversity in the stressed fish was significantly lower than that in the control. Statistically significant differences were observed in the Chao1 (676.65 ± 133.33 versus 960.40 ± 129.87, *p* < 0.001), Shannon index (4.44 ± 0.26 versus 6.33 ± 1.21 *p* < 0.001) and Simpson index (0.85 ± 0.032 versus 0.94 ± 0.02, *p* < 0.001). Principal component analysis (PCA) revealed a separation of the two groups (**Figure 4B**). Multivariate dispersions (PERMDISP) analysis showed that the stressed fish had significantly higher distances from the group centroid than the control fish (*P* < 0.05, **Figure 4C**), suggesting that the IM community structures of stressed fish were less homogeneous than those of healthy fish. Microbial composition analysis found that ten genera, including *Prevotella*, *Cetobacterium, Coprococcus, Abiotrophia* and *Blautia* were overrepresented in stress fish, whereas ten genera, including *Bifidobacterium, Lactobacillus, Bacillus* and *Faecalibacterium*, were dominant in the control group (**Figure 4D**). Accumulatively, these findings demonstrated that chronic stress imbalanced intestinal homoeostasis in gibel carp.

### Chronic stress triggers systemic inflammation in gibel carp

**Figure 5:**
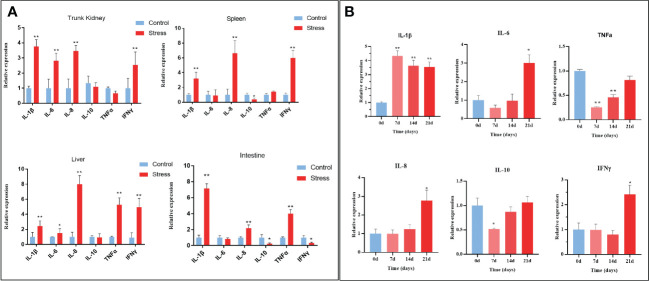
Chronic stress triggers systemic inflammation in gibel carp. **(A)** RT-qPCR analysis of inflammatory-related gene (IL-1β; IL-6; IL-8; TNFα; IFN-γ) mRNA levels in gibel carp trunk kidney, spleen, liver and intestine from healthy controls (n = 6) and stressed individuals (n = 6). **(B)** RT-qPCR analysis of inflammatory-related gene (IL-1β; IL-6; IL-8; TNFα; IFN-γ) mRNA levels in gibel carp trunk kidney from healthy controls (n = 6) and stressed individuals (n = 6) in different time points. All graphs show the means ± SEMs for individual fish, and significant differences from the control are indicated by asterisks (*P < 0.05, **P < 0.01). Blue represents heathy control and red represents stressed fish.

Increasing evidence suggests a strong link between stress and inflammation (38). Systemic inflammatory activity is typically evaluated by testing proinflammatory cytokines, including IL-1β, IL-6, IL-8, and TNF-alpha or C-reactive protein (CRP), in humans. To confirm whether chronic stress evokes systemic inflammation in fish, the expression levels of inflammation-related cytokines including IL-1β, IL-6, IL-8, IL-10, TNFα, and IFNγ were measured in the trunk kidney, spleen, intestine, and liver tissues of gibel carp. As shown in **Figure 5A**, stressed fish exhibited significantly higher levels of IL-1β, IL-6, IL-8, and IFNγ in the detected tissues than the control fish at the end of stress. In detail, the expression level of IL-1β in the trunk kidney increased from 7 d after stress and was maintained at a relatively high level during the stress period (**Figure 5B**). Other proinflammatory cytokines, including IL-6, IL-8, and IFNγ, were found to upregulated only at the end of stress. However, different expression profiles of IL-10 and TNFα were observed in diverse tissues from stressed fish. The expression levels of IL-10 in stressed fish were decreased in the spleen and intestine while showed no change in the trunk kidney and liver when compared with those in control fish. In contrast, the expression levels of TNFα in stress fish were upregulated in the live and intestine but showed no change in the trunk kidney and spleen. Notably, the increased amplitude of all detected proinflammatory cytokines remained relatively low, suggesting sustained low-grade inflammation. Altogether, chronic stress triggers systemic low-grade inflammation in gibel carp.

### Chronic stress impairs the antiviral capability in gibel carp

**Figure 6:**
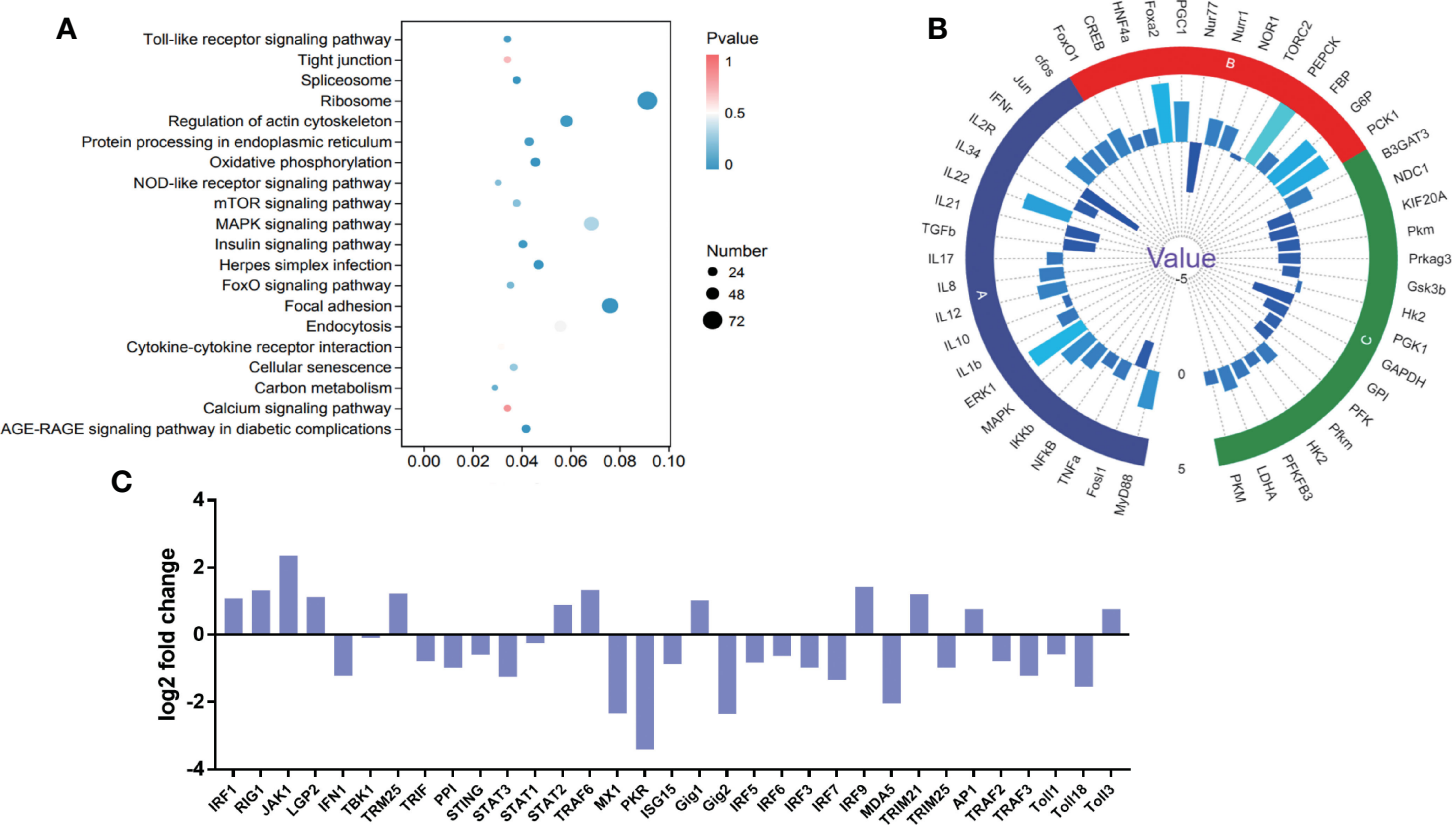
DEGs and pathway enrichment in the intestine upon chronic stress. **(A)** Dot depicting the top 20 enriched pathways based on KEGG pathway enrichment analysis of DEGs. The pathway enrichment statistics were performed by Fisher’s exact test, and those with a corrected *P* value < 0.05 were considered the most significant pathways. **(B)** Fold changes in the indicated genes related to intestinal homeostasis after CyHV-2 infection. The results are presented as the ratio of DEGs from stressed fish compared with healthy controls. Significantly, many DEGs were related to inflammation (Cluster A), glycolysis-related genes (Cluster B) and gluconeogenesis-related genes (Cluster C). **(C)** The different catalogues of DEGs in antiviral genes.

To explore the potential significance of chronic stress on immune dysfunction in stressed fish, RNA sequencing was conducted to analyse the differential gene expression in the kidney between the stressed and control groups. A total of 21,970,4336 clean reads were obtained. Transcriptome analysis revealed that 6068 genes were differentially expressed between stress and control fish, including 2728 upregulated genes and 3358 downregulated genes. KEGG enrichment analysis indicated that the DEGs were predominantly enriched in Toll-like receptor signaling pathway, viral infection, inflammation, and other metabolic pathways shown in **Figure 6A**. Significantly, many DEGs related to inflammation (Cluster A), glycolysis-related genes (Cluster B) and gluconeogenesis-related genes (Cluster C) were observed, further verifying the aforementioned findings (**Figures 5**, **6B**). In addition to the aforementioned inflammatory factors, IL-12, IL-17, and IFN-γ were activated by chronic stressors. Likewise, gluconeogenesis-related genes were significantly upregulated including Nur1, NOR1, and PEPCK. Glycolysis-related genes, including PFK, GP1, and HK2, were significantly downregulated. Moreover, IRF3, IRF7, IFN1, MX1, and other DEGs related to the antiviral innate immune response were significantly downregulated (**Figure 6C**). These observations suggested that chronic downregulated antiviral innate response.

**Figure 7:**
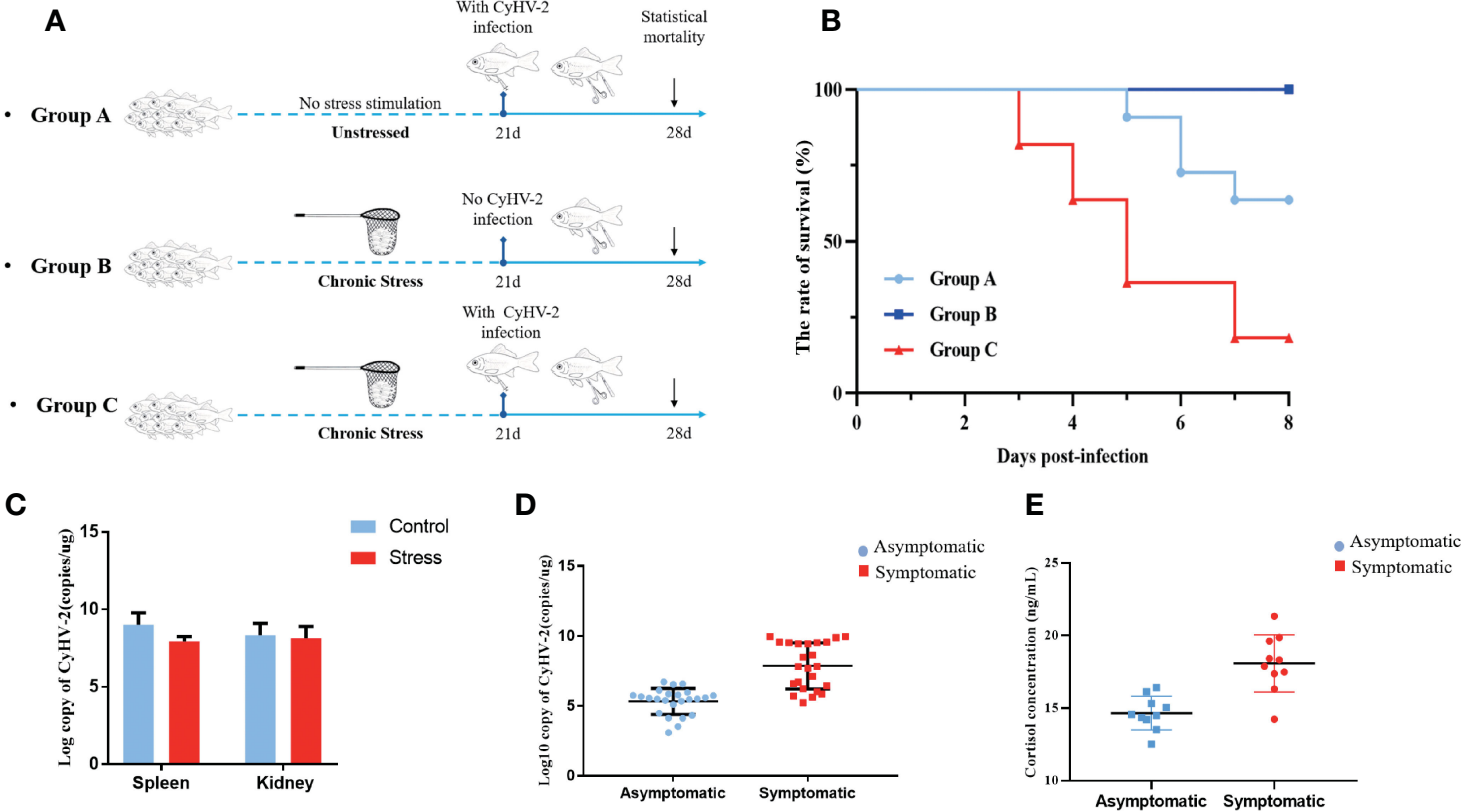
Chronic stress compromises the antiviral innate immunity of gibel carp. **(A)** Schematic representation of the viral infection schedule. Group A, unstressed fish infected with CyHV-2. Group B, stressed fish with mock infection. Group C, stressed fish infected with CyHV-2. **(B)** Survival curve of gibel carp upon CyHV-2 infection between groups A, B and C. **(C)** RT-qPCR analysis of CyHV-2 DNA copies from moribund fish with or without stress according to the standard curve based on the series of diluted plasmids containing the DNA polymerase gene. The results were expressed as copies per µg of total DNA (copies/µg DNA). **(D)** RT-qPCR analysis of CyHV-2 DNA copies from moribund fish and asymptomatic individuals collected from ponds. **(E)** ELISA analysis of serum cortisol levels in gibel carp from moribund fish and asymptomatic individuals.

According to the previous studies, it was reasonable to speculate that the antiviral ability of stressed fish was compromised. Thus, CyHV-2 was used to infect stressed fish at a dose of 0.1×LD_50_ (1×10^5.5^ copies per 50 g body weight) to evaluate their antiviral ability (**Figure 7A**). In line with this speculation, higher cumulative mortality was observed in the stressed fish than in the healthy fish after they were infected with CyHV-2. No death occurred in the mock-infected stressed fish (**Figure 7B**). Virus quantification through qPCR indicated lethal viral copies in the dead individuals suggesting the causative factor of CyHV-2 (**Figure 7C**). Clinical monitoring also demonstrated that symptomatic fish that harboured lethal viral loads had higher peripheral level of cortisol than these CyHV-2 carriers with low viral loads (**Figures 7D, E**). Taken together, these findings revealed that chronic stress impairs the antiviral capability in gibel carp.

## Discussion

Stress is considered to be a major factor contributing to poor fitness in farmed fish (39). Here, gibel carp were used to simulate sustained mild stress in aquaculture practices by a variety of stressors lasting at least 21 days. Inadequate blood glucose control, imbalance of gut homeostasis, and decreased anti-virus capability were also observed in the stressed fish. These observations imply that chronic stress communicates threats from the brain to the entire organism, which may shed light on stress management in aquaculture farming.

A growing body of evidence argues that stress has been linked to perturbations in energy metabolism (2, 38, 40, 41). Stress-induced hyperglycaemia can be identified both early after stress and in the subacute phase (2). Glucose metabolism is also affected in chronic stress paradigms including chronic restraint stress (CRS) and chronic social defeat stress (CSDS) (2, 42). In line with the increased glucose-promoting effects of glucocorticoids, fish showed elevated peripheral blood glucose 7-21 days after stress due to increased gluconeogenesis in the liver. Intriguingly, decreased concentrations of insulin accompanied by decreased expression of IR and IRS were observed suggesting the suppression of the insulin signaling pathway in these stressed fish (**Figure 2**). This did not coincide with previous studies showing that chronic restraint stress (CRS) is linked to hyperglycaemia and unaltered levels of insulin in rats (43). Interestingly, the increased level of cortisol was observed only at the end of stress (**Figure 1D**), which consistent with the results showed in a rotenone-induced mouse model of Parkinson’s disease. That indicated that cortisol could reflect its accumulation in a stressor and time dependent manner. Several studies also report that acute stress is generally associated with activation of the HPA axis (i.e., increased cortisol production). Chronic stress, however, does not consistently lead to increased cortisol levels; in fact, several studies suggest that people who have experienced chronic stress have lower baseline cortisol levels and a flattening of the typical diurnal cortisol slope (44–47). While, chronic stress induced alterations in glucose-related metabolism persist beyond the time window in which direct glucocorticoid stimulation took place. Our observations in stressed fish support the notion that hyperglycaemia may be sustained without the direct glucose-stimulating effects of glucocorticoids (48). Other factors including the expression of glucose transporter (GluT1, GluT4) and disturbance of the intestinal microbiome might imply elevated blood glucose, as observed in preclinical animal models and patients on depression (49–51). Hyperglycemia was found to contribute to mitochondrial function and ER stress inflicted damage to both peripheral and central bodily systems (51, 52). Although the exact underlying mechanism of hyperglycaemia in stressed fish was not investigated in this study, medications, aimed primarily at restoring metabolic homeostasis may constitute a novel approach to manage chronic stress in aquaculture.

Stress has a profound effect on the normal function of the immune system. Low-grade inflammation is one of the primary features under stressful conditions in mice and humans (21, 53). As expected, significantly increased but low fold changes in IL-1β, IL-6, and IL-8 were observed in the tested organs including the trunk kidney, spleen, intestine, and liver from stressed fish. Our study adds to the evidence that chronic stress triggers the state of systemic inflammation in fish. The limitation of this study is the absence of determination of serous proinflammation cytokines due to unavailable antibodies. Mechanistically, activation of the HPA axis and elevated levels of stress hormones that modify intestinal permeability have been implicated in inflammatory changes (54, 55). Stress has indeed been found to increase intestinal permeability in rats, mice, and pigs (56, 57). Our findings including morphological changes in the gut as well as increased levels of LPS and iFABP supported that increased intestinal permeability allowing bacterial liposaccharides to enter blood circulation initiated the inflammatory response. Accordingly, we also demonstrated that the changed microbiota with decreased *Bifidobacterium*, *Lactobacillus*, *Faecalibacterium* and *Flavobacterium* in fish upon long-term stress was in line with the observation in other model animals, suggesting communication between the brain-gut axis through stress hormones and metabolites (58–60). Currently, there are more questions than answers between stress and inflammation in fish. For instance, how does the vagus nerve mediate inflammation since the vagus nerve plays a key role in sensing enteric conditions and in turn modulating neural transmission and hormone release. Nonetheless, gut homeostasis including the integrated intestinal barrier as well as the balanced gut microbiota is directly associated with the systemic inflammation. Given the beneficial effects on the stress response and the intestinal barrier, metabolites, probiotics, and prebiotics show promise in the antistress management in aquaculture practices.

Chronic inflammation combined with metabolically diseased microenvironments provides the ideal conditions for viral exploitation of host cells and enhanced viral pathogenesis (61). The findings from DEG analysis and infection assays provided compelling evidence that long-term stressing impaired the antiviral capability in gibel carp. Stress deteriorated CyHV-2 infection by accelerating progression as well as increasing mortality. Field investigation also showed a positive correlation of CyHV-2 copies with serum cortisol contents. Given the latent infection, stress or immunosuppression treatment could reactivate CyHV-2 and result in increased morbidity and mortality (35, 62). These findings imply that stress is a relevant risk factor for the outbreak of CyHV-2, which provides a potential approach to prevent the occurrence of CyHV-2 by coping with stress. Intercellular mechanisms and stress-related behaviour modification are proposed to interpret the causes of compromised antiviral capability (23, 63). Elevated proinflammatory activity, decreased IFN-I signalling activity in PBMCs, reduced NK cells, T-cell suppression, and many other factors were clarified as intercellular mechanisms linked to antiviral immunity (21, 64, 65). Functional annotation analysis of DEGs identified a strong signature of intercellular mechanisms including the downregulated of PPRs, MDA5 and ISGs. Stress-boosted behavioral changes most likely elicit the release of neurochemicals, including glucocorticoids and substance P, responsible for altering the production and concentration of signal molecules (66). Further research is needed to better describe the intricacies of viral infection and stress to light the development of universal approaches to control diverse aquatic viruses.

Owing to global changes, wild or farmed fish are exposed to multiple stressors that have cascading effects from molecules to the whole individual, eventually affecting the wild or farmed populations (67). The development of a chronic stressing procedure in gibel carp provided an alternative tool for stress-triggered immunity studies. For practical and ethical consideration, zebrafish and other fish models are feasible for performing large cohorts of studies to eliminate individual variations in stress response which is now considered stress resilience and susceptibility (67, 68). Given the existing evidence concerning sex differences in stress and immune responses, the natural existing unisexual population and sex conversion in fish species provide the excellent model to study related issues (69, 70) Importantly, sex control approaches are widely adapted for breeding in aquaculture, and it is essential to evaluate the sex differences in stress hormones and their consequent immune effects (71).

## Data availability statement

The data presented in the study are deposited in the National Center for Biotechnology Information(NCBI), accession number SAMN31799252/3.

## Ethics statement

The animal study was reviewed and approved by Scientific Ethic Committee of Huazhong Agricultural University (HZAUFI-2018-016).

## Author contributions

Conceptualization, JY. Investigation, JZ, CD, and LQ. Methodology, PD, MW. Writing – original draft, CD and JY. Writing – review & editing JY and LL. All authors contributed to the article and approved the submitted version.

## References

[1] Phillips R Kraeuter AK McDermott B Lupien S Sarnyai Z. Human nail cortisol as a retrospective biomarker of chronic stress: A systematic review. Psychoneuroendocrinology (2021) 123:104903. doi: 10.1016/j.psyneuen.2020.104903 33137562

[2] Kooij M. The impact of chronic stress on energy metabolism. Mol Cell Neurosci (2020) 107:103525. doi: 10.1016/j.mcn.2020.103525 32629109

[3] Tort L. Stress and immune modulation in fish. Dev Comp Immunol (2011) 35:1366–75. doi: 10.1016/j.dci.2011.07.002 21782845

[4] Schreck CB Tort L Farrell AP Brauner CJ. Biology of stress in fish. Fish Physiol (2016) 35:1–590. doi: 10.1016/B978-0-12-802728-8.00001-1

[5] Cohen S Janicki-Deverts D Doyle WJ Miller GE Frank E Rabin BS. Chronic stress, glucocorticoid receptor resistance, inflammation, and disease risk. Proc Natl Acad Sci United States America. (2012) 109:5995–9. doi: 10.1073/pnas.1118355109 PMC3341031 22474371

[6] Petitjean Q Jean S Gandar A Cte J Jacquin L. Stress responses in fish: From molecular to evolutionary processes. Sci Total Environment. (2019) 684:371–80. doi: 10.1016/j.scitotenv.2019.05.357 31154210

[7] Thorstensen MJ Turko AJ Heath DD Jeffries KM Pitcher TE. Acute thermal stress elicits interactions between gene expression and alternative splicing in a fish of conservation concern. J Exp Biol (2022) 225:jeb244162. doi: 10.1242/jeb.244162 35673877

[8] Eissa N Wang HP. Transcriptional stress responses to environmental and husbandry stressors in aquaculture species. Rev Aquac. (2016) 8:61–88. doi: 10.1111/raq.12081

[9] Martorell-Ribera J Koczan D Tindara Venuto M Viergutz T Brunner RM Goldammer T. Experimental handling challenges result in minor changes in the phagocytic capacity and transcriptome of head-kidney cells of the salmonid fish coregonus maraena. Front Vet Sci (2022) 9:889635. doi: 10.3389/fvets.2022.889635 35591870 PMC9111177

[10] Young T Walker S Alfaro A Fletcher L Symonds JE. Impact of acute handling stress, anaesthesia, and euthanasia on fish plasma biochemistry: implications for veterinary screening and metabolomic sampling. Fish Physiol Biochem (2019) 45:1485–94. doi: 10.1007/s10695-019-00669-8 31240506

[11] Franks B Ewell C Jacquet J. Animal welfare risks of global aquaculture. Sci Adv (2021) 7:eabg0677. doi: 10.1126/sciadv.abg0677 33811081 PMC11057778

[12] Herrera M Mancera JM Costas B. The use of dietary additives in fish stress mitigation: Comparative endocrine and physiological responses. Front Endocrinology. (2019) 10:447. doi: 10.3389/fendo.2019.00447 PMC6636386 31354625

[13] Magalhes C Schrama D Farinha AP Revets D Kuehn A Planchon S. Protein changes as robust signatures of fish chronic stress: a proteomics approach to fish welfare research. BMC Genomics (2020) 21:309. doi: 10.1186/s12864-020-6728-4 32306896 PMC7168993

[14] Russell G Lightman S. The human stress response. Nat Rev Endocrinol (2019) 15:525–34. doi: 10.1038/s41574-019-0228-0 31249398

[15] Rohleder N. Stress and inflammation - the need to address the gap in the transition between acute and chronic stress effects. Psychoneuroendocrinology (2019) 105:164–71. doi: 10.1016/j.psyneuen.2019.02.021 30826163

[16] Lin W Li L Chen J Li D Hou J Guo H. Long-term crowding stress causes compromised nonspecific immunity and increases apoptosis of spleen in grass carp (*Ctenopharyngodon idella*). Fish Shellfish Immunol (2018) 80:540–5. doi: 10.1016/j.fsi.2018.06.050 29964198

[17] Fast MD Hosoya S Johnson SC Afonso LO. Cortisol response and immune-related effects of Atlantic salmon (*Salmo salar linnaeus*) subjected to short- and long-term stress. Fish Shellfish Immunol (2008) 24:194–204. doi: 10.1016/j.fsi.2007.10.009 18065240

[18] Hosoya S Johnson SC Iwama GK Gamperl AK Afonso LO. Changes in free and total plasma cortisol levels in juvenile haddock (*Melanogrammus aeglefinus*) exposed to long-term handling stress. Comp Biochem Physiol A Mol Integr Physiol (2007) 146:78–86. doi: 10.1016/j.cbpa.2006.09.003 17045829

[19] Pickering AD Pottinger TG. Crowding causes prolonged leucopenia in salmonid fish, despite interrenal acclimation. J Fish Biol (2010) 30:701–12. doi: 10.1111/j.1095-8649.1987.tb05799.x

[20] Barton BA Schreck CB Barton LD. Effects of chronic cortisol administration and daily acute stress on growth, physiological conditions, and stress responses in juvenile rainbow trout. Dis Aquat Organisms (1986) 2:173–85. doi: 10.3354/dao002173

[21] Barrett TJ Corr EM van Solingen C Schlamp F Brown EJ Koelwyn GJ. Chronic stress primes innate immune responses in mice and humans. Cell Rep (2021) 36:109595. doi: 10.1016/j.celrep.2021.109595 34496250 PMC8493594

[22] Zaletel I Filipović D Puškaš N. Chronic stress, hippocampus and parvalbumin-positive interneurons: what do we know so far? Rev Neurosci (2016) 27:397–409. doi: 10.1515/revneuro-2015-0042 26751865

[23] Sainz B Loutsch JM Marquart ME Hill JM. Stress-associated immunomodulation and herpes simplex virus infections. Med Hypotheses. (2001) 56:348–56. doi: 10.1054/mehy.2000.1219 11359358

[24] Qing H Desrouleaux R Israni-Winger K Mineur YS Fogelman N Zhang CL. Origin and function of stress-induced IL-6 in murine models. Cell (2020) 182:372–87. doi: 10.1016/j.cell.2020.05.054 PMC7384974 32610084

[25] Hasan S Suhail N Bilal N Ashraf GM Zaidi SK AlNohair S. Chronic unpredictable stress deteriorates the chemopreventive efficacy of pomegranate through oxidative stress pathway. Tumour Biol (2016) 37:5999–6006. doi: 10.1007/s13277-015-4469-9 26596837

[26] Ting EYC Yang AC Tsai SJ. Role of interleukin-6 in depressive disorder. Int J Mol Sci (2020) 21:22–8. doi: 10.3390/ijms21062194 PMC7139933 32235786

[27] Fan XT Cui L Hou TT Xue X Zhang S Wang ZZ. Stress responses of testicular development, inflammatory and apoptotic activities in male zebrafish (*Danio rerio*) under starvation. Dev Comp Immunol (2021) 114:8. doi: 10.1016/j.dci.2020.103833 32818607

[28] Drago D Tnsescu MD. The effect of stress on the defense systems. J Med Life (2010) 3:10–8. doi: 10.14219/jada.archive.1950.0133 PMC3019042 20302192

[29] Leserman J. HIV Disease progression: depression, stress, and possible mechanisms. Biol Psychiatry (2003) 54:295–306. doi: 10.1016/S0006-3223(03)00323-8 12893105

[30] Yaribeygi H Panahi Y Sahraei H Johnston TP Sahebkar A. The impact of stress on body function. Excli J (2017) 16:1057–72. doi: 10.1038/s41422-022-00689-9 PMC5579396 28900385

[31] Zhang HG Wang B Yang Y Liu X Wang JJ Xin N. Depression compromises antiviral innate immunity *via* the AVP-AHI1-Tyk2 axis. Cell Res (2022) 32:897–913. doi: 10.1016/j.coviro.2018.12.008 35821088 PMC9274186

[32] Kibenge FSB. Emerging viruses in aquaculture. Curr Opin Virol (2019) 34:97–103. doi: 10.1111/jfd.12110 30711892

[33] Luo YZ Lin L Liu Y Wu ZX Gu ZM Li LJ. Haematopoietic necrosis of cultured Prussian carp, *Carassius gibelio* (Bloch), associated with cyprinid herpesvirus 2. J Fish Dis (2013) 36:1035–49. doi: 10.1016/j.cvex.2020.01.004 23617723

[34] McDermott C Palmeiro B. Updates on selected emerging infectious diseases of ornamental fish. veterinary Clinics North America Exotic Anim practice. (2020) 23:413–28. doi: 10.3390/microorganisms8030445 32327045

[35] Chai W Qi L Zhang Y Hong M Yuan J. Evaluation of cyprinid herpesvirus 2 latency and reactivation in carassius gibel. Microorganisms (2020) 8:445. doi: 10.1016/j.fsi.2020.03.056 32245260 PMC7143840

[36] Sun J Wang J Li L Wu Z Yuan J. ROS induced by spring viraemia of carp virus activate the inflammatory response *via* the MAPK/AP-1 and PI3K signaling pathways. Fish Shellfish Immunol (2020) 101:216–24. doi: 10.3389/fendo.2018.00526 32224280

[37] Suarez-Bregua P Guerreiro PM Rotllant J. Stress, glucocorticoids and bone: A review from mammals and fish. Front Endocrinol (2018) 9:526. doi: 10.1016/0026-0495(80)90119-5 PMC6139303 30250453

[38] Chiasson J Atkinson R Cherrington A. Insulin regulation of gluconeogenesis from alanine in man. Metabolism (1979) 29:810–8. doi: 10.1111/jfb.13904 6997676

[39] Sadoul B Geffroy B. Measuring cortisol, the major stress hormone in fishes. J Fish Biol (2019) 94:540–55. doi: 10.1016/j.psyneuen.2019.05.007 30667059

[40] Qin Y Jiang X Li W Li J Tian T Zang G. Chronic mild stress leads to aberrant glucose energy metabolism in depressed macaca fascicularis models. Psychoneuroendocrinology (2019) 107:59–69. doi: 10.1016/j.physbeh.2015.02.038 31108306

[41] Thompson AK Fourman S Packard AE Egan AE Ryan KK Ulrich-Lai YM. Metabolic consequences of chronic intermittent mild stress exposure. Physiol Behav (2015) 150:24–30. doi: 10.1038/ncomms5995 25711718 PMC4545746

[42] van der Kooij MA Fantin M Rejmak E Grosse J Zanoletti O Fournier C. Role for MMP-9 in stress-induced downregulation of nectin-3 in hippocampal CA1 and associated behavioural alterations. Nat Commun (2014) 5:4995. doi: 10.3109/10253890.2011.619604 25232752 PMC4199199

[43] Nirupama R Devaki M Yajurvedi HN. Chronic stress and carbohydrate metabolism: Persistent changes and slow return to normalcy in male albino rats. Stress-the Int J Biol Stress. (2012) 15:262–71. doi: 10.1037/a0018953 21992064

[44] Suglia SF Staudenmayer J Cohen S Enlow MB Rich-Edwards JW Wright RJ. Cumulative stress and cortisol disruption among black and Hispanic pregnant women in an urban cohort. Psychol Trauma. (2010) 2:326–34. doi: 10.1097/01.psy.0000221236.37158.b9 PMC3057193 21423846

[45] Cohen S Doyle WJ Baum A. Socioeconomic status is associated with stress hormones. Psychosom Med (2006) 68:414–20. doi: 10.1038/s41398-021-01570-w 16738073

[46] Astill Wright L Horstmann L Holmes EA Bisson JI. Consolidation/reconsolidation therapies for the prevention and treatment of PTSD and re-experiencing: a systematic review and meta-analysis. Transl Psychiatry (2021) 11:453. doi: 10.1037/0033-2909.133.1.25 34480016 PMC8417130

[47] Miller GE Chen E Zhou ES. If it goes up, must it come down? chronic stress and the hypothalamic-pituitary-adrenocortical axis in humans. Psychol Bull (2007) 133:25–45. doi: 10.1073/pnas.1804412115 17201569

[48] van der Kooij MA Jene T Treccani G Miederer I Hasch A Voelxen N. Chronic social stress-induced hyperglycemia in mice couples individual stress susceptibility to impaired spatial memory. Proc Natl Acad Sci United States America. (2018) 115:E10187–E96. doi: 10.1159/000101694 PMC6205456 30301805

[49] Piroli GG Grillo CA Reznikov LR Adams S McEwen BS Charron MJ. Corticosterone impairs insulin-stimulated translocation of GLUT4 in the rat hippocampus. Neuroendocrinology (2007) 85:71–80. doi: 10.1016/j.jpsychires.2016.02.002 17426391

[50] Kahl KG Georgi K Bleich S Muschler M Hillemacher T Hilfiker-Kleinert D. Altered DNA methylation of glucose transporter 1 and glucose transporter 4 in patients with major depressive disorder. J Psychiatr Res (2016) 76:66–73. doi: 10.1016/j.conb.2020.01.016 26919485

[51] Jrbrink-Sehgal E Andreasson A. The gut microbiota and mental health in adults. Curr Opin Neurobiology. (2020) 62:102–14. doi: 10.1038/nrendo.2014.22 32163822

[52] Picard M Juster RP McEwen BS. Mitochondrial allostatic load puts the 'gluc' back in glucocorticoids. Nat Rev Endocrinol (2014) 10:303–10. doi: 10.2174/092986708784872393 24663223

[53] Andreasen AS Krabbe KS Krogh-Madsen R Taudorf S Pedersen BK Møller K. Human endotoxemia as a model of systemic inflammation. Curr Med Chem (2008) 15:1697–705. doi: 10.1053/gast.2000.18152 18673219

[54] Meddings JB Swain MG. Environmental stress-induced gastrointestinal permeability is mediated by endogenous glucocorticoids in the rat. Gastroenterology (2000) 119:1019–128. doi: 10.1053/GAST.2000.18152 11040188

[55] Karin DP Leo P. Stress induces endotoxemia and low-grade inflammation by increasing barrier permeability. Front Immunol (2015) 6:223–34. doi: 10.2174/156652408784533751 PMC4432792 26029209

[56] Caso JR Leza JC Menchen L. The effects of physical and psychological stress on the gastrointestinal tract: Lessons from animal models. Curr Mol Med (2008) 8:299–312. doi: 10.1136/gutjnl-2013-305690 18537637

[57] Vanuytsel T van Wanrooy S Vanheel H Vanormelingen C Verschueren S Houben E. Psychological stress and corticotropin-releasing hormone increase intestinal permeability in humans by a mast cell-dependent mechanism. Gut (2014) 63:1293–9. doi: 10.1152/physrev.00018.2018 24153250

[58] Cryan JF O'Riordan KJ Cowan C Sandhu KV Dinan TG. The microbiota-Gut-Brain axis. Physiol Rev (2019) 99:1877–2013. doi: 10.1002/jnr.24476 31460832

[59] Peirce JM Alviña K. The role of inflammation and the gut microbiome in depression and anxiety. J Neuroence Res (2019) 97:1223–41. doi: 10.1080/19490976.2020.1869501 31144383

[60] Deng YY Zhou MF Wang JF Yao JX Yu J Liu WW. Involvement of the microbiota-gut-brain axis in chronic restraint stress: disturbances of the kynurenine metabolic pathway in both the gut and brain. Gut Microbes (2021) 13:1–16. doi: 10.1111/j.1349-7006.1997.tb00469.x PMC7872056 33535879

[61] Tsuzurahara S Sata M Iwamoto O Shichijo S Kojiro M Tanikawa K. Detection of MAGE-4 protein in the sera of patients with hepatitis-c virus-associated hepatocellular carcinoma and liver cirrhosis. Cancer Science. (1997) 88:915–8. doi: 10.1016/j.fsi.2020.05.020 PMC5921520 9369941

[62] Wei C Kakazu T Chuah QY Tanaka M Kato G Sano M. Reactivation of *cyprinid herpesvirus 2* (CyHV-2) in asymptomatic surviving goldfish *Carassius auratus* under immunosuppression. Fish Shellfish Immunol (2020) 103:302–9. doi: 10.1002/smi.2508 32439507

[63] McVicar A Ravalier JM Greenwood C. Biology of stress revisited: intracellular mechanisms and the conceptualization of stress. Stress Health (2014) 30:272–9. doi: 10.1038/nm.3589 23868544

[64] Heidt T Sager HB Courties G Dutta P Iwamoto Y Zaltsman A. Chronic variable stress activates hematopoietic stem cells. Nat Med (2014) 20:754–8. doi: 10.1016/j.cmet.2021.03.006 PMC4087061 24952646

[65] Shen L Hu P Zhang Y Ji Z Shan X Ni L. Serine metabolism antagonizes antiviral innate immunity by preventing ATP6V0d2-mediated YAP lysosomal degradation. Cell Metab (2021) 33:971–87. doi: 10.3390/v14051115 33798471

[66] Goswami P Ives AM Abbott ARN Bertke AS. Stress hormones epinephrine and corticosterone selectively reactivate HSV-1 and HSV-2 in sympathetic and sensory neurons. Viruses (2022) 14:1115. doi: 10.1016/j.scitotenv.2022.156910 35632856 PMC9147053

[67] Shahjahan M Islam MJ Hossain MT Mishu MA Hasan J Brown C. Blood biomarkers as diagnostic tools: An overview of climate-driven stress responses in fish. Sci Total Environ (2022) 843:156910. doi: 10.3390/cells10071630 35753474

[68] Dziedzicka-Wasylewska M Solich J Korlatowicz A Faron-Górecka A. What do the animal studies of stress resilience teach us? Cells (2021) 10:1630. doi: 10.1038/s41467-021-27187-7 34209787 PMC8306023

[69] Gao A Su J Liu R Zhao S Li W Xu X. Sexual dimorphism in glucose metabolism is shaped by androgen-driven gut microbiome. Nat Commun (2021) 12:7080. doi: 10.1016/j.biopsych.2019.04.028 34873153 PMC8648805

[70] Hodes GE Epperson CN. Sex differences in vulnerability and resilience to stress across the life span. Biol Psychiatry (2019) 86:421–32. doi: 10.1007/s11427-021-2075-x PMC8630768 31221426

[71] Li XY Mei J Ge CT Liu XL Gui JF. Sex determination mechanisms and sex control approaches in aquaculture animals. Sci China Life Sci (2022) 65:1091–122. doi: 10.1016/S0006-3223(03)00323-8 35583710

